# Metabolomics and health: from nutritional crops and plant-based pharmaceuticals to profiling of human biofluids

**DOI:** 10.1007/s00018-021-03918-3

**Published:** 2021-08-19

**Authors:** Andrey S. Marchev, Liliya V. Vasileva, Kristiana M. Amirova, Martina S. Savova, Zhivka P. Balcheva-Sivenova, Milen I. Georgiev

**Affiliations:** 1 Department Plant Cell Biotechnology, Center of Plant Systems Biology and Biotechnology, 4000 Plovdiv, Bulgaria; 2 Laboratory of Metabolomics, Department of Biotechnology, The Stephan Angeloff Institute of Microbiology, Bulgarian Academy of Sciences, 4000 Plovdiv, Bulgaria

**Keywords:** Metabolomics, Crops, Medicinal plants, Biofluids, Biomarkers

## Abstract

During the past decade metabolomics has emerged as one of the fastest developing branches of “-omics” technologies. Metabolomics involves documentation, identification, and quantification of metabolites through modern analytical platforms in various biological systems. Advanced analytical tools, such as gas chromatography–mass spectrometry (GC/MS), liquid chromatography–mass spectroscopy (LC/MS), and non-destructive nuclear magnetic resonance (NMR) spectroscopy, have facilitated metabolite profiling of complex biological matrices. Metabolomics, along with transcriptomics, has an influential role in discovering connections between genetic regulation, metabolite phenotyping and biomarkers identification. Comprehensive metabolite profiling allows integration of the summarized data towards manipulation of biosynthetic pathways, determination of nutritional quality markers, improvement in crop yield, selection of desired metabolites/genes, and their heritability in modern breeding. Along with that, metabolomics is invaluable in predicting the biological activity of medicinal plants, assisting the bioactivity-guided fractionation process and bioactive leads discovery, as well as serving as a tool for quality control and authentication of commercial plant-derived natural products. Metabolomic analysis of human biofluids is implemented in clinical practice to discriminate between physiological and pathological state in humans, to aid early disease biomarker discovery and predict individual response to drug therapy. Thus, metabolomics could be utilized to preserve human health by improving the nutritional quality of crops and accelerating plant-derived bioactive leads discovery through disease diagnostics, or through increasing the therapeutic efficacy of drugs via more personalized approach. Here, we attempt to explore the potential value of metabolite profiling comprising the above-mentioned applications of metabolomics in crop improvement, medicinal plants utilization, and, in the prognosis, diagnosis and management of complex diseases.

**Supplementary Information:**

The online version contains supplementary material available at 10.1007/s00018-021-03918-3.

## Introduction

**Fig. 1:**
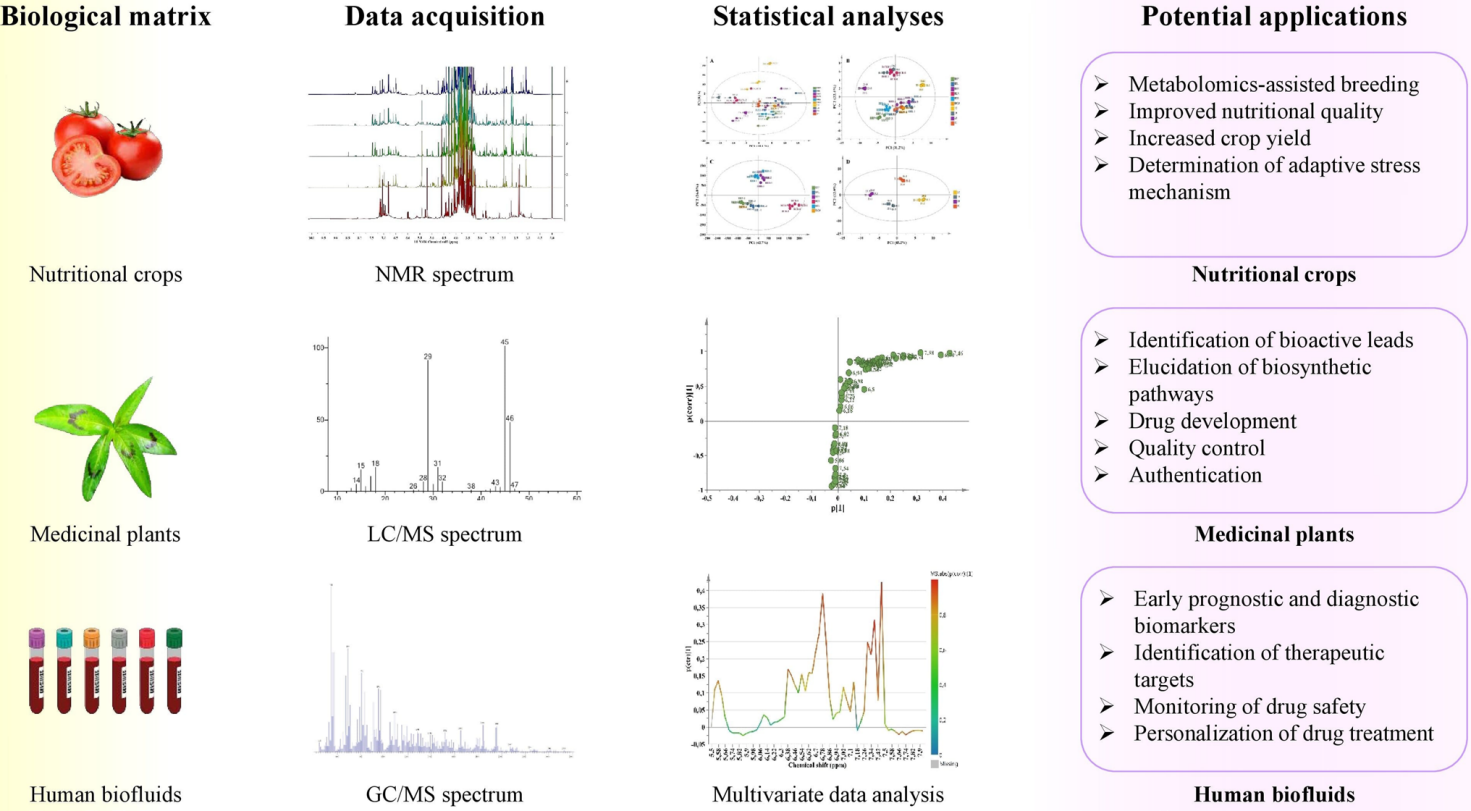
Key steps of metabolomics workflow, including selection of biological subjects, metabolite profiling, data analysis, interpretation, and potential outcomes of the integrated datasets

The metabolome has been defined not only as the total pool of all metabolites in a cell but also as a receiver of the flow of the biological information leading to the definition of a certain phenotype. Metabolites are intermediates or end-products of the metabolism. Metabolomics is a continuously developing, data-driven and high-throughput tool, whose main objective is the comprehensive identification, quantification and real-time interpretation of the primary and specialized/secondary metabolites (SMs) in a given biological system as a result of environmental and genetic interactions or due to the growth and development of an organism [1–3]. The metabolome is in constant change; therefore, metabolomics is a closer reflection of the phenotype of a cell, tissue, or an organism than the other “-omics” approaches, such as proteomics, transcriptomics, or genomics. For example, changes in messenger RNA (mRNA) are necessary for protein synthesis during transcription and the levels of proteins should be in correlation with the increased levels of mRNA. Localization of mRNA to specific subcellular compartments allows spatial regulation of gene expression that is required for polarized cell morphology and motility. The composition of RNA–protein complexes determines whether an mRNA molecule will undergo translation or be degraded. However, the translated proteins might not always be active; therefore, the alterations at a proteome level do not necessarily correlate to changes in the biochemical phenotype. Nevertheless, all these processes lead to biochemical reactions that result in alterations to metabolic pathways and metabolite pools. The biochemical phenotype of a cell or tissue can be properly defined by investigation of the metabolome when compared to gene expression [2, 3]. The quantitative and qualitative changes of the cellular metabolites correspond to the gene function, which determines metabolomics as a critical tool in systems biology and functional genomics. The metabolite composition is in direct correlation with the cell functional status as determined by its environment, which means that metabolomics attempts to measure the metabolome changes in a biological system as a response to a challenge to its normal homeostasis (Fig. 1). Therefore, metabolomics attempts to fill the gaps between genotype and end-phenotypes [1, 4]. These challenges might originate not only from the environment [5] but also from physiological changes, pathological or infectious diseases, or interaction with drugs and other external stimuli [6]. Therefore, metabolomics has potential to target many subjects for applications across several fields, such as chemotaxonomy, nutrition, pharmacology, and personalized medicine [7–9]. In plant science, metabolomics has been adopted to study not only the metabolite profiles of different crops or medicinal plants but also the taxonomic or biochemical differences of plant species grown in different ecotypes [5] or to trace the response of the metabolites under biotic/abiotic stimuli and plant adaptation [10]. Other applications include studying the difference between wild and domesticated or transgenic plants, crop improvement [11], biosynthesis pathway elucidation, correlation between metabolic markers and biological activity of medicinal plants [12], quality control of herbal preparations, and others [13]. In human and animal studies, metabolomics has become a cornerstone for understanding the difference between physiological and pathological state through development of specific biomarkers [14]. Any metabolite concentration that has abnormal deviation (higher or lower concentration) could be a sign of dysfunctional or a perturbed metabolic pathway, indicating the presence of a disease. The identification of the qualitative and quantitative metabolite changes between healthy and abnormal tissue could potentially aid recognition of the suspected disease, explain its causes, and contribute to its treatment [15]. Therefore, the outcomes of such large-scale metabolomics analyses could be useful in the diagnosis of a disease, identification of therapeutic targets, detection of disease-specific markers and offer solutions for prevention, monitoring of drug efficacy, and safety [16]. Usually, alterations in metabolic profiles are monitored by analyzing biofluids, such as blood, urine, and saliva or analyzing tissues to find out the origin, behavior, or eventual outcome of the disease [17, 18].

Metabolomics studies can be performed via untargeted metabolite profiling (referred to as metabolic fingerprinting) or through targeted metabolite profiling. Untargeted (discovery-based) metabolomics is a hypothesis-generating approach that enables global detection of all metabolites in a biological sample, giving a broader snapshot of the whole metabolome and the linked phenotypes. This approach aims to detect as many compounds as possible and further, by applying a statistical analysis, to observe the patterns of metabolites across samples. These patterns allow the classification of the samples into one or more groups of biological significance giving information about the relative quantification of the metabolites. On the other hand, targeted (validated-based) metabolomics is focused on a predetermined set of compounds related to a metabolic pathway of interest or specific groups of metabolites. Unlike metabolic fingerprinting, targeted metabolomics includes the possibility of absolute quantitation of the metabolite levels. This approach is usually chosen when testing a prior hypothesis or used for validation of the metabolites identified during untargeted analysis. While targeted and untargeted approaches might be used together, untargeted metabolomics is expected to be more widely used because of its scanning pattern and ability to collect vast amounts of data in a short time, giving it an advantage in discovering biomarkers or elucidating metabolic profiles [19, 20].

In the current review we attempt to perform a brief summary of the most frequently used analytical tools for metabolite profiling and metabolomics and their potential application in metabolome studies in plants and mammalians. Regarding plant science, examples giving an overview of the influence of plant environment, plant growth, development, and metabolite alterations corresponding to the changed gene architecture are presented. The application of metabolomics in biomarker discovery for nutritional and crop yield improvement based on abiotic/biotic stress or pathogens stimuli is given. The utilization of metabolomics for defining biomarkers predicting bioactivity of medicinal plant extracts, as well as biomarkers for authentication and quality control assessment of plant pharmaceuticals, is highlighted. Finally, the implementation of metabolomics in disease prognosis, diagnosis, and treatment is discussed, based on the metabolite differentiation analyzed in biofluids in healthy individuals and patients with established pathology.

## Metabolomics platforms

The two main platforms used for metabolite profiling are based on nuclear magnetic resonance (NMR) spectroscopy or mass spectrometry (MS), usually performed in a hyphenated mode with one or several separation technologies, including gas chromatography (GS) or liquid chromatography (LC) [19–21]. The main characteristics of these analytical platforms are presented in Table 1. The choice of an analytical approach depends on its selectivity, accuracy, precision, speed, and sensitivity—no single technique is capable of identifying the large variety of chemical structures and properties of the total metabolome of a given biological system. Therefore, the available analytical techniques are very often used in combination, since they can complement each other regarding the preferential coverage of diverse types of metabolites. Furthermore, the integration of contemporary two (2D)- and three (3D)-dimensional approaches within metabolomics experiments provides a broader perspective for interpretation of the obtained data [3–5].

**Table 1:** Comparison of frequently employed analytical platforms in metabolomics [7, 22]

Analytical platform	Applications	Advantages	Disadvantages
GC/MS	Hydrophobic and polar compounds with low molecular weight Ionization method: electron impact (EI)	High sensitivity (10^–12^ M) Accuracy (< 50 ppm) Mass range (< 350 Da) Suitable for volatile compounds Available databases	Destructive Requires derivatization Unsuitable for non-volatile compounds Relatively long run time Multiple derivatization of certain compound classes are possible
LC/MS	Polar compounds and SMs Ionization method: atmospheric pressure chemical ionization (API) and electrospray ionization (ESI)	High sensitivity (10^–15^ M) Accuracy (50–100 ppm) Mass range (< 1500 Da)	Destructive Low separation of LC column Specific retention times Specific chromatographic conditions
NMR	Exploits the ability of spin active nuclei to absorb and re-emit pulsed electromagnetic radiation of a characteristic frequency pattern when placed in a magnetic field; provides information about molecular structure, chemical environment and molecular motion	Non-destructive Highly reproducible Accurate quantification Minimal need of sample preparation Analysis of wide range of chemical structures	High capital cost of instrumentation Low sensitivity (10^–6^ M) Overlap of metabolite signals in 1D spectra

### MS-based metabolomics platforms

#### GC/MS

Gas chromatography–mass spectrometry has been widely applied as a high-throughput analytical tool for metabolite profiling with a high rate of sensitivity. It offers highly reproducible fragmentation patterns, separation, detection, and identification due to the electron impact (EI) ionization point of supply. The mass spectrometer measures the mass-to-charge (m/z) ratio of molecular ions or molecular ion fragments [3, 10]. The advantages of GC/MS are high sensitivity, precision, resolution, and reduced running costs, as well as, the wide availability of protocols, retention times, and mass spectra data availability in many libraries. However, the use of GC/MS is limited to thermally stable and volatile compounds, making the analysis of high molecular weight compounds (> 1000 Da) difficult. A derivatization step is required to make compounds volatile, but some compounds might stay underivatized. The metabolites typically detected by GC/MS are mainly associated with tricarboxylic acid (TCA) cycle, glycolysis, urea cycle, amino acid metabolism, and fatty acid metabolism. Therefore, GC/MS can be undoubtedly used to explore the dysregulation of neurotransmitter, hormones, and purine metabolism in different neurological diseases [22]. Different mass analyzers, such as single quadrupole (Q), triple quadrupole (QqQ), ion trap (IT), and time of flight (TOF), can be coupled to the GC. However, GC–Q–TOF/MS is frequently preferred, because of the fast scan times, improved deconvolution, and high mass accuracy. The separation efficacy and the number of separated and identified compounds could be elevated by the application of a 2D GC/GC–TOF–MS [23, 24].

#### LC/MS

Higher mass primaries and SMs (< 1500 Da) are detected by targeted and untargeted tools such as LC/MS. In contrast to GC/MS, in LC/MS the sample does not require prior preparation and the components are separated in a liquid phase [25]. Ultra-performance liquid chromatography (UPLC) and high-performance liquid chromatography (HPLC) have been frequently used for metabolite separation using different eluting columns, such as reverse phase, ion exchange, and hydrophobic interaction columns in accordance with the chemical properties of the metabolites [23, 26]. After separation, the metabolites in the sample are ionized, typically by soft ionization techniques, such as electrospray ionization (ESI) or atmospheric pressure chemical ionization (API), and often utilizing both positive and negative ion generation and detection modes. Other ionization sources, such as desorption electrospray ionization (DESI) and matrix-assisted laser desorption ionization (MALDI), have been applied to achieve higher-resolution imaging. Many types of MS, including Q, QqQ, IT, or TOF, have been used depending on the sensitivity, mass resolution, and range required. For instance, MALDI-assisted TOF/MS is suitable for accurate and quantitative metabolite profiling at the single-cell level, detection of low mass protein (with a mass range 1–300 kDa) with a high sensitivity of approximately 10^–18^ M [13]. This allows LC/MS to detect high molecular weight metabolites, which are polar and thermo-labile with very high sensitivity. A major disadvantage of this analytical platform is the difficulty to establish large mass spectral libraries and many research groups have their own “in-house” libraries [1, 15, 20, 26]. Along with targeted and untargeted metabolomics, using LC/MS is possible to perform dynamic multiple reaction monitoring (MRM)-based pseudo-targeted metabolomics and quantification and parallel reaction monitoring (PRM)-based larger-scale targeted metabolomics quantification. These are two newly emerged strategies, both of which can measure a large number of metabolites with reliable quantitative arrays. A compound precursor ion is isolated in Q1, fragmented in Q2, and subsequently all generated MS/MS fragment ions are monitored in parallel on a high-resolution, accurate mass, and full-scan mass spectrometer. Thus, hundreds of metabolites, such as sugars or peptides, are able to be identified in complex biological systems and give information about the physiological status of an organism due to possible perturbation of different processes, such as bile acid biosynthesis, citrate cycle, or glycolysis [27, 28].

### NMR-based spectroscopy as a metabolomics platform

The NMR-based metabolomics has several advantages compared to the MS-based platforms, since it is a rapid, non-destructive, and non-selective method that omits the need for a prior chromatographic separation or derivatization of the analytes. The NMR analysis allows for obtaining quantitative information, since the signal of the metabolites is proportional to their molar concentration [29]. In addition, the in vivo NMR can generate kinetic measurements and examine the metabolic responses in one sample. The principle of this spectroscopy is based on atomic interactions. In a strong homogeneous magnetic field, atoms with a non-zero magnetic moment (^1^H, ^13^C, ^14^N, ^15^N, and ^31^P) absorb and re-emit electromagnetic radiation, which is characterized by its frequency (chemical shift), intensity, magnetic relaxation properties, and signal splittings (*J*-couplings), all of which reflect the environment of the detected nucleus. The relaxation of these excited nuclei back to their ground state gives a specific spectrum of radiation that can be used for identification and quantification of the metabolites in a complex biological sample [30]. In addition to chemical shifts, NMR frequencies are modified by a series of couplings: spin–spin scalar couplings, which depend on covalent bonding; spin–spin dipolar couplings, which depend on internuclear distances; and for nuclear spins greater than 1/2, quadrupolar couplings between the electric field gradient at the nucleus and the charge distribution of the nucleus. These NMR interactions are anisotropic, which means that they depend on the sample orientation relative to the magnetic field direction. Because of these orientation-dependent chemical shifts, internuclear couplings, and quadrupolar couplings, NMR spectra encode 3D structural information [31]. Some limitations of NMR spectroscopy include lower dynamic range, resolution, and sensitivity, resulting in limited coverage of primary and SMs compared to MS. Another limitation is the overlap of signal spectra. However, the application of miniaturized radiofrequency coils, superconducting magnets, cryogenic probes, as well as multi-dimensional NMR techniques have contributed to overcome some of these limitations [17, 29]. For example, the combination of 2D and 3D NMR spectra with fast Fourier transformation and non-uniform sampling is expected to become standard for multi-dimensional data acquisition with complex biological macromolecules, such as membrane proteins or monoclonal antibodies [32, 33].

Some of the most frequently used 2D NMR experiments are heteronuclear single quantum coherence (HSQC), which uses magnetization transfer between nuclei, usually between hydrogen and carbon atoms; homonuclear correlation spectroscopy (COSY), which identifies spins that are coupled to one another; and total correlation spectroscopy Y (TOCSY), which cross peaks are formed for both directly and indirectly coupled nuclei. The most frequently used 3D NMR experiment is [^13^C, ^1^H] HSQC-TOCSY, which provides 3D correlations in a reduced dimensionality manner, facilitating high-resolution and unambiguous assignments [34].

The NMR-based metabolomics in solution or solid-state applications can be used for structural determinations and functional studies with possible “in-cellulo” applications. The application of NMR spectroscopy can vary from membrane proteins in biological systems to polymers and cements in chemistry, providing an opportunity to investigate the object of interest in its native form. However, in solution NMR spectra, the averaging of anisotropic NMR interactions gives rise to a series of very sharp transitions. Solid-state NMR spectra are typically very broad and featureless due to two predominant effects: direct homonuclear and heteronuclear dipolar coupling and the full effects of anisotropy. High-resolution NMR spectra can provide the same type of information that is available from corresponding solution NMR spectra, but a number of special techniques/equipment are needed, including magic-angle spinning, cross polarization, special 2D experiments, and enhanced probe electronics [31].

During the past few decades significant efforts has been devoted to extend the applications of NMR spectroscopy to large molecular systems. The characterization of the conformational properties of proteins and their interaction mode with physiological partners has recently become a major research topic for understanding biological function at molecular level. During recent years, the multi-dimensional NMR spectroscopy has become the technique of choice to obtain atomic-resolution information for proteins, and to extract useful information on the structural ensemble that the proteins forms in solution. In particular, NMR allows the identification of peptide regions with increased propensity to form *α*-helical or extended (*β*-strand) structures that often play a role in molecular recognition events, or the characterization of transient long-range interactions. Furthermore, NMR is a powerful technique to characterize binding events in terms of interaction surfaces, and to study eventual conformational transitions of the proteins upon binding to its partner(s). Some of the challenges here, such as signal overlapping and faster relaxation time, leading to poor spectral sensitivity might be overcome by reducing the number of the resonance by a proper choice of isotope-labeling schemes. The 2D NMR techniques, such as transverse relaxation optimized spectroscopy (TROSY), are exclusively selecting the slowly relaxing resonance line, eliminating the faster relaxing resonance. Thus, TROSY disregards half of the potential signal and is appropriate technique to analyze molecules larger than 15,000 Da. Along with that 2D nuclear Overhauser effect spectroscopy (NOESY) is useful in determining which signals arise from protons that are close to each other in space even if they are not bonded. The 2D NOESY is used to determine protein–ligand contacts [35].

## Metabolic profiling of nutritional crops and medicinal plants

### Nutritional crops

Metabolomics is one of the “-omics” approaches that has been extensively applied for crop improvement, since many metabolites are unique to plants and play an important role in crop yield and nutritional quality. Therefore, metabolomics is one of the primary tools for the study of abiotic stress tolerance, pathogen resistance, metabolic-assisted breeding of crops, and ecotype influence on plant growth and development, all of which entail the synthesis of a large number of metabolites [11, 19, 24, 36–57]. This type of environmental metabolomics analysis investigates the interaction between plants and their environment, aiming to explain the effect of the identified metabolites on plant adaptation [39–41]. Selected examples for the application of metabolomics in nutritional crops research are presented in Supplementary Table S1.

Biotic and abiotic stress adversely affect crop productivity and cause massive reduction in the annual crop yield. Metabolomics with other “-omics” tools is used to explain the mechanisms of plant adaptation to abiotic/biotic stress, understand the stress regulation process from genome to phenome, and to perform a selection of resistant plants with improved stress tolerance [11, 19, 24, 42–49]. The GC/MS profiling of *Oryza sativa* L. (rice) transgenic plants revealed that the elevated amount of trehalose in leaves is responsible for the increased drought, saline, and sodic tolerance. Furthermore, trehalose modulated other metabolic switches, leading to significant changes in the levels of sugars, amino, and organic acids in leaves [46]. The GC/MS profiling could also follow the influence of climate change over metabolite variations in rice. High night temperatures led to a decrease in sugar phosphates and sucrose and a higher abundance of monosaccharides in panicles, indicating impaired glycolysis and higher respiration-driven carbon losses [47]. In *Hordeum vulgare* L. (barley) the metabolites that act as potential biomarkers for abiotic stress were revealed by GC/MS profiling. The concentrations of amino acids (phenylalanine, tryptophan and tyrosine) and sugar acids, including galactaric acid and glucuronic acid, were increased in the salt-tolerant cultivar [48]. *Fagopyrum tataricum* L. Gaertn. (tartary buckwheat) is a nutritional crop with high flavonoid content and high salt sensitivity. The comprehensive analysis of the transcriptome and the metabolome of salt-treated buckwheat was able to identify the genes and metabolites responsible for salt tolerance. Genes mainly involved in secondary metabolism were upregulated, which resulted in the increased biosynthesis of rutin and suppression of carotenoid biosynthesis [49].

Metabolomic-assisted breeding provides crops varieties with improved nutritional quality and yield [3, 50, 51]. Quantitative trait locus (QTL) analyses are useful in detection of genes/loci in wild species that may improve the yield or quality-related traits in elite varieties, which combined with genome-wide associated studies (GWAS), are an efficient way to discover genes associated with phenotypes [3]. *Zea mays* L. (maize) plays a vital role in human nutrition and energy supply and has been used as a model crop for understanding useful traits with improved stress resistance, nutritional quality, growth, and development. Primary metabolites, such as carbohydrates, amino, and organic acids, determine the relevant crop quality traits related to nutritional content and composition, and are also linked to plant growth and development [44]. It is considered that the genetic diversity of maize has decreased during domestication, compared to its ancestor (teosinte), leading to a reduction of some specific nutritional and flavor qualities, due to directional artificial selection, natural selection, or genetic drift [50]. The generation of introgression populations by crossing wild relatives and modern elite cultivars is a good strategy for identifying “hidden” genes in wild species and has been widely used in maize and other crops to find the balance between high yield and quality in modern crop breeding programs. A total of 65 primary metabolites were measured using a GC–Q–TOF/MS in different tissues by untargeted metabolite profiling of a BC_2_F_7_ population, generated from a cross between a wild maize relative (*Z. mays* spp. *mexicana*) and maize inbred line Mo17. The established QTL hotspots revealed that the primary metabolites frequently increased in the presence of alleles from the wild-type genome, while the opposite was observed for grain yield and shape trait QTLs. For example, a negative correlation was observed between quinic acid and grain starch content. Eight primary metabolites, mainly amino and organic acids (2-oxoglutaric acid, arginine, glutamic acid, glutamine, isocitric acid, leucine, 3-caffeoyl-cis quinic acid, and succinic acid), had negative correlation with maize kernel starch and viscosity, although glutamate, for instance, is a biological indicator for a selection of high grain yield in maize. The GWAS analysis revealed that the major QTL accounting for 31.4% of the variation in gamma aminobutyric acid (GABA) content with the favorable allele from *mexicana* was mapped on chromosome 1, where the *ZmGAD* gene encoding glutamate decarboxylase was the most significant. The glutamic acid could be decomposed into GABA under the function of glutamate decarboxylase [24]. Furthermore, metabolomics could provide considerable insight into both genetic and biochemical regulation of metabolism by comparison of cultivated maize and the study of metabolite QTLs (mQTLs) in recombinant inbred lines [44] or diverse association mapping populations [45]. The broad-scale metabolite profiling of *Lycopersicon pennellii* (Correll) D’Arcy (tomato) fruits using a UPLC system coupled to executive Orbitrap mass detector was performed on lines heterozygous for the introgression of chromosomal segments from the *L. pennellii* genome. In this way, it was possible to evaluate the heritability mode of the mQTLs secondary metabolism, which is an important characteristic from a breeding perspective. The application of real-time quantitative PCR gave insight into the transcriptional control mechanisms of a subset of the mQTLs, including those for hydroxycinnamates, acyl-sugar, naringenin chalcone, and a range of glycoalkaloids, indicating that these compounds might increase during domestication [2]. By using broadly targeted LC–MS/MS-based metabolic profiling in tandem with GWAS and QTLs, an increase in the nutritional quality of tomatoes was achieved by reducing the presence of anti-nutritional steroidal glycoalkaloids [51].

Metabolomics is also of great interest for food quantification, including molecular based traceability and nutritional value [20, 52–58]. It was applied for the generation of metabolic markers between two species used as sources of goji berries, such as *Lycium barbarum* L. and *Lycium chinense* Mill., which are very similar red ovoid fruits and difficult to discriminate with morphological or molecular markers [20]. The application of LC–ESI–TOF/MS and GC–EI/MS discriminated between the two *Lycium* species according to chlorogenic acid, asparagine and quinic acid, which were more abundant in *L. chinense*, whereas *L. barbarum* accumulated more lycibarbarphenylpropanoids A-B, coumaric acid, fructose and glucose. Additionally, the chemometrics revealed the metabolic markers differentiating both the *Lycium* species and the *Solanum* species (*S. lycopersicum*) with lycopene, carotene, glutamate, and GABA dominating in the latter. Lycibarbaphenylpropanoids and zeaxanthin ester were characteristic metabolites for the goji fruits [20]. Metabolite profiling has been applied for the purpose of understanding the nutritional quality [52] and yield potential of *Glycine max* L. Merr. (soybean) [29]. By applying a comprehensive GC/MS and LC/MS profiling, it was established that soybean seeds sprouted by watering in the dark increases their nutritional quality. A large number of macromolecules related to energy production (*myo*-inositol, phytosterols, antioxidants, isoflavones, and soyasaponins) and metabolites associated with health benefits and/or taste quality (*myo*-inositol, isoflavone aglycones, B soyasaponins, antioxidants, and phytosterols) increased with germination time [52]. In addition, the GC/MS metabolic profiling could be used to predict the antioxidant content in black soybean, considering sucrose, threonic acid, epicatechin, procyanidin B2, and cyanidin-3-*O*-glucoside as such markers [53, 54]. The GC/MS profiling of several *Prunus avium* L. (cherry) cultivars identified the metabolic markers associated with the fruit quality. Among these were primary metabolites, such as fructose, glucose, sorbitol, and malic acid, while among the SMs the most important were quercetin-3,4-*O*-diglucoside, esculetin, rutin, and neochlorogenic acid [56]. In the era of emerging bio-based economies, renewable materials are vital for the production of biofuels and biomaterials. The multi-dimensional solid-state NMR metabolomics has been applied to lignin, cellulose, and hemicellulose quantification through direct measurement without chemical or enzymatic pre-treatment [57]. Using solid-state NMR spectroscopy revealed increased biosynthesis of cellulose and xylan in *O. sativa* mutants compared to the wild type [58].

Metabolomics is also essential in following metabolite biosynthesis during plant growth and development [49, 59, 60]. For example, the LC/MS-based profiling also gave insight into the phytochemical variations and the morphology, in particular seed color of tartary buckwheat. Flavonoids and anthraquinones were related to variations in seed color, while flavonoids in particular with the seed shape [49]. In another study, the profile of carotenoids and flavonoids during fruit development was investigated using combinatory metabolomics and transcriptomics approach in “Cara cara” navel orange. It was found that some potential transcription factors (TFs), such as Cs3g19420, Cs3g23270, Cs5g26720, Cs7g11810, and Cs7g26660, might positively regulate important pathway genes from the carotenoid and flavonoid biosynthesis. Flavonoids and especially narirutin were accumulated in the flesh at the early stages 60 days after florescence and the TFs could potentially bind to phenylalanine ammonia-lyase (*PAL*), 4-coumarate-CoA ligase (*4CL*), chalcone synthase (*CHS*), and chalcone isomerase promoters (*CHI*), respectively. The transcripts of five carotenogenesis genes, including 1-deoxy-d-xylulose-5-phosphate synthase, deoxyxylulose 5-phosphate reductoisomerase, geranylgeranyl diphosphate synthase, phytoene synthase, and lycopene *β*-cyclase were highly correlated with lycopene contents [59]. The addition of abscisic acid and a biostimulant SUNRED at the pre-véraison stage of grape ripening significantly changed the expression of key genes and their enzyme activities from the anthocyanin pathway. Some of the early biosynthesis genes, such as *PAL*, *CHS*, and *CHI*, had an increased expression during the whole process. However, the late biosynthesis genes, e.g., dihydroflavonol 4-reductase, UDP-glucose:flavonoid-3-*O*-glycosyltransferase, as well as the myeloblastosis (MYB) 1 and MYB2, increased at the post-mature period. The gene expression of flavanone 3-hydroxylase, flavonoid 3′5′-hydroxylase, and anthocyanidin synthase were upregulated during the early-to-mid period of fruit ripening [60].

By investigating the metabolite profile of nutritional crops, it would be possible not only to enrich its phytochemical characterization but would also facilitate the choice of an optimum harvest time and balanced nutritional quality of the plant. Sugar metabolism was identified to be the crucial metabolic and transcriptional component that differentiated floral organ in rice and determined its reproductive success under stress. Some important metabolites for this process were sucrose, glucose-6-P, fructose-6-P, glucose, maltose, and *myo*-inositol accompanied as with high expression of genes encoding a sugar transporter and a cell wall invertase [61].

In summary, metabolomics has been developed as a powerful tool for evaluating phenotypic variance within broad genetic populations. In an attempt to fill the gap between genomes and end-phenotypes, the analysis of differences in metabolite abundance has also proved to be an efficient strategy. Metabolomics provides considerable insight into both genetic and biochemical regulation of metabolism, with studies including the comparison of cultivated crops and their wild cultivars. The obtained information might be useful in evaluating the heritability mode of the metabolites responsible for the nutritional quality and crop yield, which is an important characteristic to study from a breeding perspective. In addition, an understanding of the mechanisms of plant adaptation to abiotic/biotic stimuli and the response in primary or SMs as a consequence is the base for selection of biomarkers, resulting in crop improvement, extending the breeding portfolio beyond the traditional improvement targets of oil and protein to a wide variety of chemical compounds, including essential amino acids, vitamins, antioxidants, and other metabolites of physiological and nutritional importance.

### Application of metabolomics in metabolic engineering of plants

Engineering plant metabolic pathways is not always an easy task, which arises from a lack of precise understanding of the entire network of genes, transcripts, proteins, and metabolites in biological systems. In spite of that, the fundamental approaches, such as introduction/suppression of single or multiple genes encoding rate-limiting enzymes or side branches pathway, seem to be very effective in regulation of metabolites biosynthesis. Metabolite profiling and comparative compositional analysis revealed the metabolic changes in response to gene modification in transgenic rice cultivars. Disruption of *OsSULTR3*;*3* resulted in a reduction of phytic acid in the transgenic rice, leading to its nutrient composition improvement. Phytate is poorly digestible by monogastric animals and phytic acid may interact with essential microelements in the intestinal tract, such as zinc, thus making them poorly available. Hence, phytic acid is widely regarded as a key anti-nutrient in feed for monogastric animals, as well as, in food for humans [62]. The biosynthesis of anthocyanins is regulated by the interaction of DNA binding of MYB, MYC (encoding basic helix-loop-helix (bHLH)), and WD40 TFs and occurs when all of them form an active WBM complex. Genetic and some abiotic factors, e.g., drought, temperature, light, and plant growth regulators, affect the expression of these TFs and the related structural genes and as a consequence the biosynthesis of anthocyanins itself [63]. Under low or high nitrogen levels the anthocyanins biosynthesis is orchestrated by the elevated expression of VvMYBA1, VvMYB5b, and VvLBD39 TFs, which can induce the early and late biosynthetic genes from the anthocyanins biosynthetic pathway [64].

In a pair-wise transcript-to-metabolite analysis, it was found that the induction of PgMYB2 TF in *Panax ginseng* C.A. Mey by methyl jasmonate (MeJA) resulted in increased biosynthesis of ginsenosides in the roots through the upregulation of dammarenediol synthase [65]. Five TFs, including three MYBs, one ethylene-responsive, and one bHLH, were found to be candidate regulators of benzylisoquinoline alkaloid biosynthesis in *Nelumbo nucifera* [60]. Global transcriptome analysis identified the MeJA-responsive R2R3-MYB TF-encoding the gene *SmMYB1* in *Salvia miltiorrhiza* Bunge. Overexpression of *SmMYB1* significantly promoted phenolic acid accumulation and upregulated the expression of genes encoding key enzymes in the phenolic acid biosynthesis pathway, including cytochrome P450-dependent monooxygenase [66].

Large-scale metabolite profiling assays have allowed researchers to access the global datasets of metabolites and their respective metabolic pathways. The integration of metabolomics with transcriptomics and genetic modification has established new avenues for studying the fine-tune mechanisms relevant to crop improvement or biosynthesis of targeted SMs. The effective combination of these approaches is the guiding point to investigate the functional genes and the characterization of the metabolites in order to prioritize the candidate genes for downstream analyses and offers specific markers indicative for improved commercially important traits or potential biological activity of medicinal plants.

### Plant-based pharmaceuticals

The chemical machinery of plants provides an immense number of diverse and complex structures, especially among the SMs, that generate interest as bioactive leads with potential benefits for human health. An impressive number of modern drugs are based on plant-derived pharmaceuticals, such as taxol from *Taxus brevifolia* Nutt, artemisinin from *Artemisia annua* L., metformin from *Galega officinalis* L., vincristine, and vinblastine from *Catharanthus roseus* L. G. Don [67–69]. Currently, the ethnopharmacology-based drug discovery process starts with an evaluation of the claimed effect on health of the crude plant extract aiming at identification of a specific compound or fraction (group of compounds) responsible for the actual biological response. The phytochemical characterization of medicinal plant extracts is often targeted at specific marker compounds that are typical major SMs. However, these major compounds are not always the ones that carry the biological activity of the extract. Identification of plant-derived bioactive compounds is laborious and time consuming with an overall low success rate. In this regard, plant metabolomics is an advantageous holistic approach towards the chemical characterization of natural extracts [13, 69].

The combination of hyphenated techniques, such as high-resolution MS with NMR-based metabolite fingerprinting, provides precise information about both qualitative and quantitative chemical composition of a crude plant extract [70–75]. Consequently, untargeted metabolite profiling contributes to accelerated identification of plant-derived SMs. Integration of metabolomics data with bioassays results shortens the drug discovery process through assisting the bioassay-guided fractionation (biochemometrics approach). Additionally, plant metabolomics is an indispensable tool for defining or refining pathway structure [73, 74], increasing specific SMs production through metabolic engineering [75], as well as assuring the quality and safety of plant-derived natural products [76–79]. Selected studies representing the application of metabolomics in medicinal plant research are presented in Supplementary Table S2.

Revealing the dynamic of accumulation of valuable bioactive SMs throughout the plant growth phases is of great importance for the determination of harvest time and assertion of quality control. For instance, prenylated flavonoids, such as epimedin A, epimedin B. epimedin C, and icariin, are believed to be the primary bioactive components in *Epimedium pubescens* Maxim. During leaf growth and maturation two major dynamic trends were displayed: epimedin A and epimedin B, which gradually increased and accumulated mainly after reproductive stages, especially at harvest stage and prenyl-flavonoids, such as baohuoside I and II, were generally higher at the full flowering stage and then decreased greatly at later growth stages [78]. However, using 3D LC/MS framework is giving new frontiers for imaging the spatial distribution of small molecules in plant tissue. In this respect atmospheric-pressure 3D-surface matrix-assisted laser desorption/ionization mass spectrometry imaging (3D-surface MALDI MSI) was used to investigate plant chemical defense at the topographic molecular level for the *Asclepias curassavica* L. It was established that the mechanical damage stimulated the secretion of defense metabolites, the dominant of which were cardiac glycosides [79].

Untargeted metabolite profiling facilitates the acquisition of detailed chemical characterization of a selected plant extract, as well as comparison within a group of plants of interest aiming at exposure of compounds with certain biological activity. In this regard, a metabolomics study compared the profiles of the following medicinal plants *Aloysia triphylla* Royle, *Apium graveolens* L., *Coriandrum sativum* L., *Laurus nobilis* L., *Lavandula officinalis* Chaix, *Marrubium vulgare* L., *Mentha spicata* L., *Inula viscosa* L. Aiton, *Petroselinum crispum* (Mill.) Fuss, *Salvia officinalis* L., and *Thymus vulgaris* L. by means of ^1^H NMR. The applied metabolomics approach identified several flavonoids (apigenin derivatives, apiin, catechin, genistein, quercetin, etc*.*), organic acids (ferulic, chlorogenic, *p*-coumaric acids), trigonelline, forsythoside, and rosmarinic acid as SMs, as well as certain primary metabolites. The phytochemical characteristics of the studied extracts were further correlated with the plants antioxidant and cytotoxic potential on SK-N-BE(2)-C neuroblastoma and HepG2 hepatocarcinoma cell lines. The phytochemical analysis and the observed biological response revealed *T. vulgaris* and *M. spicata* as the most promising sources of bioactive compounds (such as rosmarinic acid) to counteract oxidative stress [80]. *Symphytum offcinale* L. is a medicinal plant with known local analgesic and anti-inflammatory potential commonly characterized with the presence of allantion, rosmarinic, ellagic, and caffeic acids, as well as high content of polysaccharides. However, the mentioned compounds used alone do not produce the same biological effect as the extract. More comprehensive investigation of its metabolome through combinatorial NMR and ESI–MS approach aiming at bioactivity-guided fractionation has identified novel SMs, such as comfreyn A that contribute to the anti-inflammatory effect of the extract [12]. Metabolite profiling of *Alpinia oxyphylla* Miq. crude ethanolic extract coupled with bioactivity assay of zebra fish Parkinson’s disease model have led to isolation of novel lead oxyphylla A through bioactivity-guided fractionation [81]. Comprehensive metabolite profiling via UHPLC–TOF/MS of *I. viscosa* leaf extract revealed a diverse profile of phenolic compounds, mainly derivatives of kaempferol-*O*-(feruloyl)-hexoside and quercetin-*O*-*p*-coumaroyl-*O*-hexoside, which were not described previously. In addition, the authors have provided biological data for the effect of *I. viscosa* extract on cell viability and intracellular redox status in several human cell lines (HaCaT keratinocyte cell line, SH-SY5Y neuroblastoma cell line, HepG2 cell line, and HCT 116 colorectal carcinoma cell line) that was further analyzed with the metabolomics results, hence suggesting the studied extract as a source of potent phytochemicals [82]. Metabolomic profiles of several medicinal plants with known sedative effects, including *Valeriana officinalis* L., *Melissa officinalis* L., *Hypericum perforatum* L., and *Passiflora incarnata* L., were analyzed through GC/MS and LC–qTOF/MS and correlated with brain-derived neurotrophic factor (BDNF) expression in neuroblastoma cell line. Secondary metabolites corresponding to high BDNF expression were identified from the groups of flavonoids, xanthones, coumarines, tannins, naphthalenes, terpenoids, and those with a carotenoid skeleton [83].

Metabolite profiling is utilized in the authentication, quality, and safety control of medicinal plants. Typhae pollen, the dry pollen of Typhaceae plants (*Typha angustifolia* L., *Typha orientalis* C. Presl, or the plants of same genus), is commonly used in the traditional Chinese medicine for cardiovascular problems, such as *angina pectoris,* or for stroke prevention. Untargeted metabolite profiling through UHPLC–Q–TOF/MS in combination with chemometrics of the following standard markers isorhamnetin-3-*O*-(2G-*α*-l-rhamnosyl)-rutinoside, umbelliferone, kaempferol, isorhamnetin-3-*O*-neohesperidoside, and astragalin was proposed as a combinatorial strategy for quality control of natural products and crude plant material of Typhae pollen. The authors provided sufficient data on validation that the proposed combinatorial markers assure successful discrimination between crude Typhae pollen and commercial natural products that contain it [75]. Authentication and safety control of natural products of *Polygonum multiflorum* Thunb. was assessed via UHPLC–Q–Orbitrap/MS analysis. Data of the metabolite profiling was correlated with toxicity assays which resulted in the characterization of torachrysone-*O*-hexose and emodin-8-*O*-glucoside as hazardous compounds that could be utilized as potential toxicity markers [76].

Metabolite fingerprinting could be used to discriminate different species of the same genus. For example, untargeted metabolite profiling via rapid-resolution LC–ESI–QTOF/MS was successfully implemented to classify and characterize seven *Lonicera* species [84]. Similarly, ESI–QTOF–MS/MS was used to develop dereliction and quantitation approach to comprehensively characterize *Salvia* species [85]. Another approach to authenticate and discriminate closely related species is the heteronuclear multiple-bond correlation of the NMR-based metabolite profiling, termed as 2D NMR barcoding. Such an approach was used to successfully distinguish three medicinal *Glycyrrhiza* species with close metabolite profiles especially regarding their flavonoid and chalcone metabolomes [68]. A metabolomics approach evaluated simultaneously the genetic variations in several populations of *Verbascum* species, revealing significant variability in the content of harpagoside (major bioactive compound), phenolics, and essential oils among the tested samples. Such data could be utilized in metabolomics-assisted breeding to obtain a plant with desired genotype [74]. In another study, purple-leaf tea cultivars of *Camellia sinensis* L. Kuntze were subjected to untargeted metabolite profiling through UPLC–Q–TOF/MS and gene expression analysis. The integrated metabolomics and genomics data identified *4CL*, *ANS*, and *UFGT* genes in the anthocyanin biosynthetic pathway and the *HEME* gene in the chlorophyll biosynthetic pathway as involved in the high anthocyanin level and low chlorophyll level in the purple-leaf tea species [86].

Collectively, metabolomics is a substantial part of modern ethnopharmacological research. The considerable datasets generated from the metabolite profiling are used in bioactive leads finding, biosynthetic pathways elucidation, authentication safety, and quality evaluation of plant-derived natural products. Moreover, integration of metabolomics with biochemometrics and/or other “-omics” tools aids bioactivity-guided fractionation and drug discovery. Metabolomics can trace the alteration induced as a result of the interaction between a plant-based pharmaceutical and the homeostatic system in a living organism.

## Human biofluids profiling in health and disease

Metabolic phenotyping of clinical biological samples, such as human biofluids or tissues, is based on the fundamental paradigm that any local or systemic disruption in the homeostasis will be reflected in substantial changes of system parameters. Discrimination between the metabolite profile of a control cohort and a group diagnosed with certain pathology would ideally result in early prognostic or diagnostic marker identification or individual response to therapy predictions among other potential benefits [87]. Shifts in metabolic phenotypes in tissue compartments and biofluids are a consequence of gene–environment interactions; hence, integration of metabolomics with complementary “-omics” data would provide even stronger information for disease course prognosis and identification of relevant biomarkers [22]. Metabolic profiling of human biofluids permits high-throughput generation of molecular fingerprints and a wide range of pathological conditions have been evaluated with it including cardiovascular diseases, neurodegenerative conditions, metabolic disorders, infections, and certain types of cancer [7, 87–89]. Pharmacometabonomics is the term used to describe the application of metabolomics for predicting the individual response to therapy [90, 91].

Different types of biomarkers, such as plasma, blood, urine, or saliva based, have been applied in order to define the most prominent metabolite disorders or alterations typical for heart failure (HF) [88, 91], type 2 diabetes (T2D) symptoms [92], or to predict the efficacy of drug treatment [90]. Numerous metabolic screening strategies have been developed to measure the chemical diversity of a population’s biofluids with the aim to provide clinicians, medical scientists, and epidemiologists with a clearer picture of the presence and severity of cardiovascular disease, prognosis, and response to treatment. The most common clinical biomarkers used are relatively limited to troponin and brain natriuretic peptide, dependent on the damage to the heart muscle, or myocyte “stretch,” respectively [88]. Metabolic impairment is an important contributor to HF pathogenesis and progression. A targeted tandem MS/MS assay was used to detect 63 metabolites in fasting plasma, revealing novel circulation metabolites, such as long-chain acylcarnitine (LCAC), which reflected impaired or dysregulated fatty acid oxidation that is independently associated with HF. The LCACs are intermediates in the fatty acid *β*-oxidation pathway. Although typically short-lived, LCAC accumulates in states of inefficient fatty acid oxidation (FAO), which may be attributed to defects in mitochondrial FAO enzymes or increased FAO relative to TCA flux; this leads to a bottleneck of carbon substrates at the TCA cycle [91]. The NMR spectra of urine samples revealed that the biomarkers typical for T2DM were glutamine, uric acid, and asparagine, which are part of the purine/pyrimidine pathway [87]. In another study an increase of the branched amino acids (leucine, isoleucine and valine), non-esterified fatty acids (palmitic acid, stearic acid, oleic acid, and linoleic acid), and lysophosphatidylinositol species (16:1, 18:1, 18:2, 20:3, 20:4, and 22:6) were considered as biomarkers of T2D in Chinese population [92]. Very often saliva is the preferred body fluid for metabolic studies due to its non-invasive collection method. The LC–MS/MS applied profiling of saliva revealed that differences could be detected in diverse groups of metabolites, such as alterations in the concentrations of steroids, alkaloids, neurotransmitters, and hormones [93]. Another non-invasively available biofluids is the skin sebum. Lipid-like structures and small molecules were detected in sebum samples from PD patients through a LC/MS-based metabolomics approach. The authors have elucidated metabolites belonging to ceramide, triacylglycerol, and fatty acyl classes as downregulated and glycosphingolipid and fatty acyl metabolites as upregulated in the PD group compared to the controls, hence suggesting their implementation as useful PD biomarkers [94].

Metabolite profiling of human biofluids and tissue samples is utilized to predict the individual response to a certain treatment regimen. For instance, the NMR-based metabolomics was applied to predict the chemotherapy efficacy of drugs towards metastatic breast cancer. The baseline levels of formate and acetate were identified as predictive markers, which revealed that the gemcitabine-carboplatin could achieve clinical benefits compared to patients with higher levels of these markers [90]. Proton NMR metabolomics identified the biomarkers associated with the effects of induction of chemotherapy in locally advanced head and neck squamous cells carcinoma. The molecular response to chemotherapy involved an increase of the serum lipids, which was accompanied by the simultaneous decrease of alanine, glucose, and *N*-acetyl-glycoprotein. These molecules were found to significantly correlate with the regression of the primary tumor [95]. The ^1^H NMR-based metabolomic approach has been used to obtain informative metabolic snapshots of GL261 glioma cells acquired at different time points during glabrescione B (GlaB) treatment. It was found that GlaB stimulated the glycolytic metabolism in glioma, increasing lactate production. The high glycolytic rate could in part support the cytotoxic effects of GlaB, since the simultaneous blockade of lactate efflux with *α*-cyano-4-hydroxycinnamic acid affected glioma cell growth [96]. ^1^H NMR spectroscopy can be used as a tool to monitor the cell response to different constraints, *e.g.,* irradiation treatment of tumors. After gamma irradiation procedure of breast cancer was found that the signals of neutral lipids and glutamine signals are increased [97]. Using 2D and 3D spectra from solid-state NMR spectroscopy is possible to observe the protein assembly in cell membranes and also to observe any structural changes due to drugs acceptance or pathological stimuli [98]. Isotope labeling combined with a 2D solid-state NMR was used to discriminate the structure of misfolded *α*-synuclein amyloid fibrils, which are the principal components of Lewy bodies and neurites, hallmarks of Parkinson’s disease [99]. The use of ^13^C isotope is crucial to study the hallmarks of metabolic reprogramming in cancer and to assess the associations between metabolic pathway preferences and other cell autonomous processes. This targeted metabolic study is important for the therapeutic strategy. The use of [U–^13^C] glucose and [U–^13^C] glutamine were applied to study the nutrient consumption and metabolism in lung cancer cells. The high glucose consumption correlates with lactate secretion, while glutamine consumption correlated with glutamate secretion, corresponding to the elevated expressions of the genes lactate dehydrogenase A and glutaminase, respectively [100]. The employed 3D metabolomics by LC/MS and NMR spectroscopy revealed the mechanism-specific inhibitory profiles of amifostine (a clinically used drug with a radioprotective and cytoprotective properties to normal tissues in patients subjected to anti-cancer therapies) against vascular endothelial growth factor A (VEGF-A) and deferoxamine-induced angiogenesis. The most prominent molecular pathways for the pro-angiogenic factors clearly inhibited by amifostine were aspartate and asparagine metabolism, urea cycle/amino group metabolism, and purine metabolism [101]. The 2D Orbitrap secondary ion MS and liquid extraction surface analysis-tandem MS were performed directly on brain tissue sample. This approach could predict the corresponding pathways for tumor relapse, of which tryptophan, linoleate, cytochrome, phenylalanine, and tyrosine metabolism were the most affected [102]. The combination of high spatial resolution of secondary ion mass spectrometry (under 200 nm for inorganic species and under 2 mm for biomolecules) with the high mass-resolving power of an Orbitrap (> 240,000 at *m/z* 200) allows exogenous and endogenous metabolites to be visualized in 3D with subcellular resolution. This could be applied to detect and identify metabolites with subcellular spatial resolution and valuable for studying diseases, as well as to provide fundamental biological insights into metabolism heterogeneity at the single-cell scale, e.g., brain tissue [103]. High-resolution MALDI-MS has been used for simultaneous analysis of drugs and drug metabolites with endogenous biomolecules in cancer cells, and distinguishes potential markers of therapeutic response. It has also been applied to study the metabolite differences between normal and tumor brain tissue. The most prominent difference was found in the upregulated fatty acid metabolism [104]. Isotope labeling and LC/MS analyses revealed that creatine–phosphagen ATP-recycling system is a major mechanosensitive target responsible for the pancreatic cancer cell environment changes. This system depends on arginine flux through the urea cycle, which is reflected by the increased incorporation of carbon and nitrogen from l-arginine into creatine and phosphocreatine on stiff matrix [105]. Aspartate provided high correlation (81.4%) for a biomarker metabolite identified in serum with adenocarcinoma lung cancer, while pyrophosphate as a metabolite identified in plasma revealed 77.9% correlation with the disease. However, the best performance was achieved using a combination of 8 metabolites (maltose, maltotriose, cysteine, 3-phophoglycerate, citrulline, pyrophosphate, tryptophan, adenosine-5-phosphate) in plasma classifier resulting in an accuracy of 77.3% [106]. A panel of 7 metabolites (uracil, histamine, cysteine, 3-hydroxypicolinic acid, uric acid, indoleacrylic acid, and linoleic acids) were classified as early-stage biomarkers for lung adenocarcinoma [107]. The GC- and LC/MS platforms used for liver metabolic profiling revealed that metabolites belonging to TCA cycle (malic, fumaric, succinic acids), glycolysis, purines, and lipid (glycerol 3-phosphate, glycerylphosphorylethanolamine, glycerophosphocholine) metabolism, had high correlation with hepatocellular carcinoma progression [108].

Solution NMR-based metabolomics and the opportunities to use isotope labeled atoms has extended its applicability as time-resolved NMR monitoring of important biological processes, such as post-translational modifications of proteins or RNA modifications [35]. The continuous NMR measurements of the tRNA along its maturation route is possible through the introduction of isotope labeled RNAs. Due to the non-destructive nature of NMR and the use of cryoprobes, it is possible to directly monitor RNA modification events in a continuous and time-resolved fashion in a single sample. Through the atomic-resolution information provided by NMR spectroscopy multiple RNA modifications introduced to the same substrate, e.g., methylations on nearby nucleotides, can be easily distinguished. The monitoring is based on the fact that imino signals of RNAs are very sensitive to their chemical environment and the imino groups carried by uridines and guanosines are easily observed in ^1^H-^15^N correlation spectra, called a direct effect. However, this effect is followed by another indirect effect, which enables to detect modifications on adenosines and cytosines, even though they do not carry imino groups. Thus, using 2D (^1^H-^1^H)-NOESY, 2D (^1^H-^15^N)-BEST-TROSY, and standard 2D (^1^H-^15^N) HSQC experiments were used to characterize and understand the dynamic regulation of modification circuits in tRNAs [35].

Post-transcriptional protein modifications, including phosphorylation, acylation, alkylation, and glycosylation, could be also studied by 2D ^1^H-^15^N HSQC, ^1^HN-^1^H TOCSY, and ^1^H-^13^C HSQC NMR spectroscopy. The study of these modifications is crucial in understanding the biological functions of these proteins, as well as cellular signaling processes such as cell–cell communication, cell growth, and differentiation, mediating intracellular transport and initiating programmed cell death. For instance, lysine acetylation has a key role in the regulation of gene expression through the modification of core histone tails by histone acetyltransferases. It is also important for DNA repair, p53 functions, and microtubule stabilization [109]*.*

**Fig. 2:**
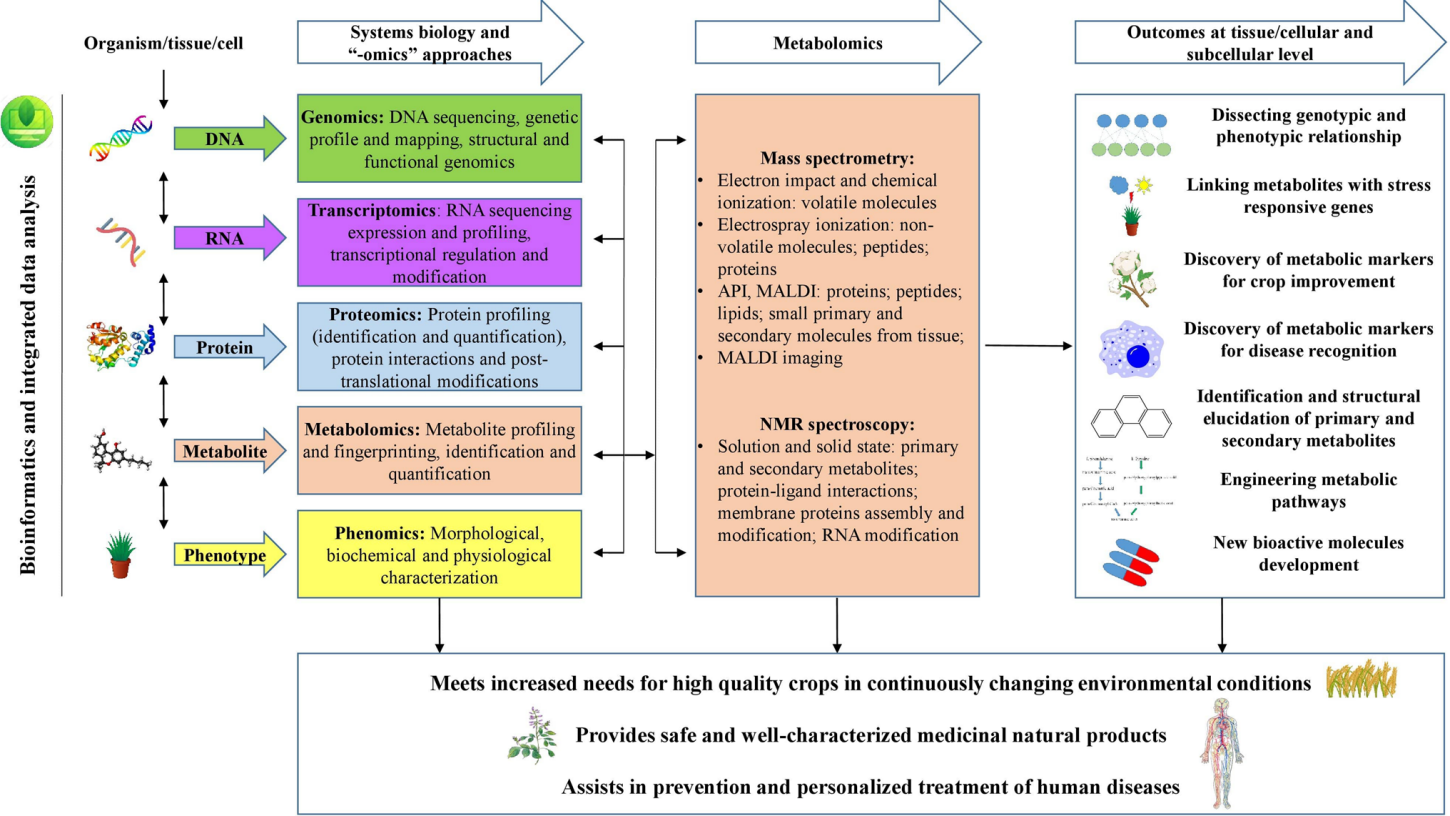
Schematic view of “-omics” technologies, mainly metabolomics, its applications, and contributions in terms of nutritional crops improvement, medicinal plant characterization, disease prevention and treatment, and improved human health

Plants are important sources of food, medicines, and industrial raw materials. Understanding the diversity, functions, and pathways of the plant-derived natural metabolites is fundamental not only for food security and nutrition but also to produce novel pharmaceuticals and food supplements through plant metabolic engineering. On the other hand, understanding the biological responses to various dietary or herbal-derived molecules, including biohazards and active herbal components, is vital for human health. Metabolomics can be particularly useful in crop improvement in terms of yield, quality, and safety, and applied to increase crop production in order to meet the needs of food supply for the increasing population. In addition, plant metabolomics helps better understand biochemical bases of dietary foods consumption, how metabolites change along the process of plant growth and development and the production, storage, and transportation of plant-derived supplements. Furthermore, plant metabolomics aids in the discovery of novel natural product-based drugs, the quality evaluation of herbal medicines, and pharmaceutical production, thus benefiting human health, mainly through prevention of diseases, such as obesity, cardiovascular diseases, and cancer, among others. Metabolomics of human biofluids is a useful platform with various applications in the context of precision medicine. It is employed to identify the hallmarks of different diseases and to aid their prognosis, diagnosis, and treatment, through identification of therapeutic targets. A model presenting the link between the applications of metabolomics in plants and humans is presented in Fig. 2.

## Conclusions and future perspectives

Metabolomics has taken an important place in plant and human biology exploration and turned into a reliable system for documentation of vast datasets developing robust biomarkers with different application. Metabolomics along with transcriptomics has been applied in plant research for identification of candidate genes functions that govern the entire biological apparatus and establish the link between genotype and phenotype in response to several environmental factors, such as climate conditions, ecotype differences, and abiotic/biotic stress conditions. Metabolomics combined with mQTLs and GWAS can discriminate the biomarkers responsible for crop nutritional quality and yield, thus properly discovering the way for crop enhancement in metabolomics-assisted breeding and managing the challenge for “zero hunger” expected from the Food and Agriculture Organization over the forthcoming decades. Therefore, one future direction would be to combine metabolomics with post-genomics in order to investigate genetic procedures for plants in response to their metabolism. Along with that, single cell-based metabolomics research should be employed to gain insight into cell or tissue linked with the environment response at the metabolic level.

Metabolomics is a rapidly evolving science, already well established in the field of medicine, pharmacology, and personalized medicine. Discrimination between health and disease with specific early biomarkers, prediction of disease course, and outcome are some of the most explored characteristic features of metabolite profiling in clinical practice. Furthermore, pharmacometabonomics as specialized branch of metabolomics has emerged as a tool for prediction of individualized therapy response. However, still many of clinical studies are limited in sample size, have poorly defined control groups and lack of evident validation of the candidate biomarkers in independent populations, which could be the future outlook of metabolomics in clinical settings. Another future perspective might be the non-invasive probing of metabolites within a single live cell. This could be very useful in mapping subcellular metabolites, understanding cell behavior, and cellular and subcellular mechanisms, identifying the possible drug sensitivity and phenotyping different cancer cell types. Although single-cell metabolomics lags behind other “-omics” methods for the lack of proper toolsets for non-perturbative and targeted detection (mainly due to the very small size of the cells and low concentrations of the metabolites), it has the potential to offer deep insights on the metabolic reprogramming that accompanies many diseases and open up avenues for clinical translation. Along with these, an additional challenge is the establishment of appropriate databases and personnel training.

Owing to the multi-dimensional exploitation of small-molecule metabolite databases and the progression of untargeted metabolomics studies, most metabolomics studies provide phenotype data. However, due to challenges associated with the uncertain identities of differential metabolites and unavailability to obtain purified compounds there appeared difficulties leading to inadequate functional metabolomics compared to phenotypic metabolomics. Additionally, this barrier impeded the translational applications of metabolomics in life sciences. Therefore, the utilization of targeted metabolomics as a general tool to overcome the limitation of conventional phenotypic metabolomics in combination with related “-omics” and biochemical techniques must be a new strategy in functional metabolomics for studying the associated mechanisms of the dysregulated small-molecule metabolism present in different biological systems.

### Supplementary Information

Below is the link to the electronic supplementary material.

Supplementary file1 (DOCX 29 kb)

## Data Availability

Not applicable.
