# Supplementary material for: Metabolomics and health: from nutritional crops and plant-based pharmaceuticals to profiling of human biofluids
Source: Cell Mol Life Sci. 2021 Aug 19;78(19-20):6487–503. doi: 10.1007/s00018-021-03918-3 (PMC8558153; doi:10.1007/s00018-021-03918-3)
Supplement: Supplementary file 1 — Supplementary file1 (DOCX 29 kb) [file 18_2021_3918_MOESM1_ESM.docx]

Supplementary Table S1. Selected examples for application of metabolomics in nutritional crops improvement.

| Crop | Analytical platform | Sample tissue | Metabolic traits | Main findings | Metabolite profiling | Reference |
| --- | --- | --- | --- | --- | --- | --- |
| *Brassica rapa* L. (turnip) | NMR  LC/MS | Leaves | Primary and secondary metabolites | Nutritional quality evaluation following biochar application | Untargeted | [40] |
| *Citrullus* spp. (melon and watermelon) | UPLC-ESI-MS/MS | Fruits | Primary and secondary metabolites | Evaluation of the phylogeny on fruit flesh chemical composition | Untargeted | [38] |
| *Eriobotrya japonica* Lindl (loquat) | LC-ESI-MS/MS | Fruits | Primary and secondary metabolites | Evaluation of metabolic differences between white- and yellow-fleshed loquat fruit related to taste | Targeted | [39] |
| *Fagopyrum tataricum* L. Gaertn. (tartary buckwheat) | UPLC-MS/MS | Seeds | Polyphenols (flavonoids and anthraquinones) | Metabolomics-assisted breeding  Relationship between secondary metabolites and morphological variations | Targeted | [49] |
|  | GC-TOF/MS | Seeds | Primary and secondary metabolites | Salinity-stress resistance | Untargeted | [10] |
| *Glycine max* (L.) Merr. (soybean) | GC-TOF/MS | Seeds | Primary and secondary metabolites | Metabolomics-assisted breeding | Untargeted | [53] |
|  | GC/MS  LC/MS | Sprouts | Primary and secondary metabolites | Nutritional and taste quality evaluation during germination | Untargeted | [52] |
|  | GC/MS | Seeds | Isoflavones and free fatty acids | Storage stability | Targeted | [41] |
|  | NMR | Seeds | Primary and secondary metabolites | Metabolomics-assisted breeding | Untargeted | [29] |
|  | NMR | Seeds and leaves | Primary and secondary metabolites | Stress tolerance | Untargeted | [55] |
|  | LC/MS | Seeds | Primary and secondary metabolites | Correlation between metabolites and antioxidant activities | Untargeted | [54] |
| *Hordeum vulgare* L. (barley) | GC/MS | Seeds | Amino acids, sugars, sugar derivatives and organic acids | Metabolomics-assisted breeding  Metabolic markers for salt-tolerant crop selection | Untargeted | [48] |
| *Oryza sativa* L. (rice) | GC/MS | Seeds and leaves | Proline, threonine, trehalose, valine, aspartate, oleic acid, palmitic acid, and vanillic acid | Increase in yield potential and abiotic stress resistance | Untargeted | [47] |
|  | GC-EI-TOF/MS | Leaves and panicles | Primary and secondary metabolites | Increase in yield potential and abiotic stress resistance | Untargeted | [46] |
|  | LC-ESI-MS/MS | Hull | Flavonoids | Increase in anthocyanin-biosynthesis | Targeted | [26] |
|  | GC/MS | Seedlings | Primary and secondary metabolites | Nutritional quality evaluation | Untargeted | [62] |
|  | GC/MS  UPLC-MS/MS | Fruits | Primary and secondary metabolites | Nutritional quality improvement | Untargeted | [57] |
| *Prunus avium* (L.) L. (cherry) | UPLC-MS/MS  GC/MS | Fruits | Primary metabolites | Metabolomics-assisted breeding directed to improved nutritional quality | Untargeted | [56] |
| *Solanum lycopersicum* L. (tomato) | UPLC-MS | Fruits | Secondary metabolites and whole metabolome | Metabolomics-assisted breeding directed to improved nutritional quality | Untargeted | [2] |
|  | UPLC-ESI-TQ/MS  GC-EI/MS | Fruits | Carotenoids, phenolic compounds and primary metabolites | Determination of nutritional quality biomarkers | Targeted | [20] |
|  | ESI-MS/MS | Leaves | Amino acids and carbohydrates | Drought stress tolerance | Untargeted and targeted | [51] |
| *Solanum lycopersicum* L. (tomato) and *Lycium* spp. (goji) | UPLCESI-MS/MS | Fruits | Carotenoids, phenolic compounds and primary metabolites | Determination of nutritional quality biomarkers | Targeted | [20] |
| *Solanum tuberosum* L. (potato) | ESI-MS/MS  GC-TOF/MS | Leaves | Amino acids and carbohydrates | Drought stress tolerance | Untargeted and targeted | [42] |
| *Triticum aestivum* L. (wheat) | NMR | Leaves and spikes | Amino acids, organic acids and sugars | Meabolic-assisted breeding  Drought-tolerant crop selection | Untargeted and targeted | [37] |
|  | NMR | Grain | Amino acids, carbohydrates, phenolic acids, and carboxylic acid | Yield and protein quality evaluation | Untargeted and targeted | [11] |
| *Triticum turgidum* L. subsp. *durum* (Desf.) Husn (durum wheat) | GC/MS | Leaves and kernels | Primary metabolites | Yield and nutritional quality improvement | Untargeted | [37] |
| *Zea mays* L. (maize) | GC/MS | Roots | Terpenoids (β-selinene and β-costic acid) | Resistance to pathogens | Untargeted | [44] |
|  | GC/MS | Leaves and kernels | Primary metabolites | Yield and nutritional quality improvement | Untargeted | [45] |
|  | GC-TOF/MS | Leaves and kernels | Primary and secondary metabolites | Yield and stress tolerance improvement | Untargeted | [50] |

Supplementary Table S2. Selected examples of application of metabolomics in medicinal plants analysis.

| Medicinal plant | Analytical platform | Sample tissue | Metabolic traits | Application | Metabolite profiling | Reference |
| --- | --- | --- | --- | --- | --- | --- |
| *Actaea racemosa* L. | HPLC-TOF-ESI/MS | Rhizomes | Marker compounds such as cimifugin derivatives, triterpene glycosides and alkaloids | Identification of markers to provide authentication of the plant and avoid toxicity or adulteration | Targeted | [8] |
| *Alpinia oxyphylla* Miq. | LC-MS/MS | Fruits | Metabolite profiling of crude extracts and fractions | Metabolomics integrated with bioactivity-guided fractionation | Untargeted | [81] |
| *Arctium lappa* L. | NMR  GC/MS  UPLC-Q-TRAP/MS | Roots | Metabolite profiling of samples from control and plants grown under copper-induced stress | Elucidating the metabolic pathways involved in copper induced stress in *A. lappa* | Untargeted and targeted | [72] |
| *Catharanthus roseus*  (L.) G.Don | MALDI-MS | Petals | Secondary metabolites | Semi-quatitative determination of vinka alkaloids distribution in *C. rosues* petals | Targeted | [69] |
| *Epilobium angustifolium* L. | RP-HPLC-PDA-  ESI/MS | Aerial parts | 20 identified secondary metabolites with oenothein B found to be the most abundant one | Correlation between bioactivity and bioavailability | Untargeted | [70] |
| *Frutillaria* spp. | UPLC-Q-TOF-MS | Bulbs | Secondary metabolites | Evaluation of candidate marker compounds for quality control and authentication of herbal products | Targeted | [77] |
| *Glycyrrhiza glabra* L. | NMR | Roots | Flavonoids | Structure-abundance-activity relationships of designated bioactivity | Targeted | [68] |
| *Salvia miltiorrhiza* Bunge | UPLC-ESI-MS/MS | Roots | Marker compounds such as danshensu, salvianolic acid A, salvianolic acid B, cryptotanshinone and protocatechualdehyde | Structure-abundance-bioactivity relationships | Targeted | [71] |
| *Symphytum*  *ocinale* L. | LC-ESI-FT/MS  NMR | Roots | 20 secondary metabolites identified | Novel bioactive lead comfreyn A (lignin) was isolated and chemical profiling was analysed in tandem with bioassay | Untargeted | [12] |
| *Verbascum songaricum* Schrenk | HPLC-PDA/MS | Aerial parts | Phenolics (rutin, rosmarinic acid, 3,4-dihydroxybenzoic acid, ferulic acid, 2,5-dihydroxybenzoic acid, salicylic acid, p-coumaric acid, ferulic acid, quercetin) and harpagoside | Evaluation of *V. songaricum* as potential source of valuable pharmaceuticals, mainly harpagoside | Targeted | [74] |
| *Zataria multiflora* Boiss. | GC/MS | Essential oil | Carvacrol, thymol, linalool, p-cymene, γ-terpinene, and α-pinene | Chemical composition and environmental factors interaction | Untargeted | [73] |
| *Arctium minus* L., *Carduus*  *nutans* L., *Cirsium vulgare* Savi, *Digitalis purpurea* L., *Senecio*  *jacobaea* L., *Verbascum blattaria* L. and *Verbascum thapsus* L. | LC-TOF-MS/MS | Roots | Primary and secondary metabolites | Evaluation of the relationship between growth rate and nutrient-storing metabolites in different types of plants | Untargeted | [25] |
